# Submucosal Invasion and Ulceration in Early Gastric Cancer Affect Mucosal Impedance

**DOI:** 10.1002/deo2.70434

**Published:** 2026-09-23

**Authors:** Kaede Miyashiro, Yohei Sawada, Hidezumi Kikuchi, Shintaro Goto, Tadashi Yoshizawa, Saki Nakano, Taka Asari, Tatsuta Tetsuya, Daisuke Chinda, Hirotake Sakuraba

**Affiliations:** ^1^ Department of Gastroenterology, Hematology, and Clinical Immunology Hirosaki University Graduate School of Medicine Aomori Japan; ^2^ Department of Anatomic Pathology Hirosaki University Graduate School of Medicine Aomori Japan; ^3^ Division of Endoscopy Hirosaki University Hospital Aomori Japan

**Keywords:** claudin, endoscopic submucosal dissection, endoscopy, gastric cancer, mucosal impedance

## Abstract

**Objectives:**

To investigate early changes in gastric cancers that are difficult to evaluate using endoscopic imaging through the assessment of mucosal impedance (MI) in endoscopic submucosal dissection (ESD) specimens.

**Methods:**

In this single‐center prospective cohort study, we included patients who underwent ESD for early gastric cancer (EGC). MI was measured at both the main tumor site and the surrounding non‐neoplastic mucosa in resected specimens. Histopathological evaluation and immunohistochemical staining for claudin‐3, ‐4, and ‐18 were performed. Furthermore, multivariate analysis was used to investigate factors influencing MI‐L.

**Results:**

The MI measured under high‐frequency current (MI‐H) of the non‐neoplastic mucosa was significantly higher than that of the tumor (*p* = 0.0025). Additionally, the MI measured under low‐frequency current (MI‐L) was significantly lower in pT1b cases than in pT1a cases (6.9 Ω vs. 9.7 Ω, *p* = 0.021). Similarly, the MI‐L was significantly lower in the pUL1 cases than in the pUL0 cases (7.5 Ω vs. 9.7 Ω, *p* = 0.030). Multivariable linear regression analysis demonstrated that, among the explanatory variables—including tumor invasion, ulcer status, macroscopic type, and the H‐scores for each claudin‐ only the H‐score of claudin‐18 was identified as an independent associated factor.

**Conclusions:**

In resected specimens, MI‐L was independently associated with claudin‐18 expression and correlated with submucosal invasion, ulcer status, and macroscopic type. MI has the potential to detect non‐surface changes involving tight junction proteins in EGC that cannot be observed via endoscopy.

**Trial Registration**: N/A.

## 1 Introduction

Electrical impedance (EI) can be used to evaluate biological tissues. Several studies have reported the detection of breast cancer using EI tomography [1, 2, 3] and skin cancer using EI spectroscopy [4, 5]. Additionally, mucosal impedance (MI) in gastric cancers has been shown to be significantly lower than that in non‐neoplastic mucosa [6].

The recently developed tissue conductance meter (TCM) measures MI using alternating currents at two frequencies, enabling assessment of low‐frequency impedance (MI‐L) and high‐frequency impedance (MI‐H), which reflect the paracellular and transcellular pathways, respectively. Studies using this device in gastroesophageal reflux disease (GERD) and functional dyspepsia (FD) showed significantly lower esophageal MI in GERD and lower duodenal MI in FD than in controls [7, 8]. In tumors, MI‐H has been shown to correlate with cell density [9]. Although MI‐L in tumors has not been extensively studied, the expression of tight junction proteins has been investigated. Notably, tight junction proteins can be used to determine the differentiation of gastric mucosa, such as gastric “claudin‐18” and intestinal claudins “claudin‐3 and ‐4,” like gastric and intestinal mucins [10]. Claudin‐3 and ‐4 expression in early gastric cancer (EGC) was significantly higher than that in gastric mucosa without intestinal metaplasia, whereas claudin‐3 expression was significantly lower at the submucosal invasive front than in the mucosal region of gastric cancer specimens [11]. Claudin‐18 expression was also significantly downregulated in gastric cancer compared with surrounding normal gastric mucosa or intestinal metaplasia [12].

The evidence from these studies shows that MI can be used to evaluate inflammation and cell density, which are difficult to assess using endoscopic imaging. We hypothesized that inflammation, tight junction disruption, and increased cell density decrease MI‐L and increase MI‐H in EGC. Thus, in this study, we aimed to investigate early changes in EGC that are difficult to assess with endoscopic imaging using TCM.

## 2 Methods

### 2.1 Study Design and Patients

This single‐center prospective cohort study was conducted at Hirosaki University Hospital between January 2023 and September 2024. Patients who underwent endoscopic submucosal dissection (ESD) for gastric neoplasms were included. Cases diagnosed with non‐gastric cancer and those in which accurate measurements could not be obtained, such as due to device measurement failure or measurements performed at non‐tumor sites, were excluded. Lesions with tumor diameters < 10 mm were also excluded, as accurate measurements may be limited in small tumors because the electrical current may not adequately pass through the lesion. Clinical data, including age, sex, use of proton pump inhibitors (PPI) or potassium‐competitive acid blockers (PCAB), resection time, *Helicobacter pylori* infection, smoking habit, drinking status, and histopathological findings based on the Japanese Classification of Gastric Carcinoma, were collected from electronic medical records.

### 2.2 Impedance‐measuring Device

**FIGURE 1:**
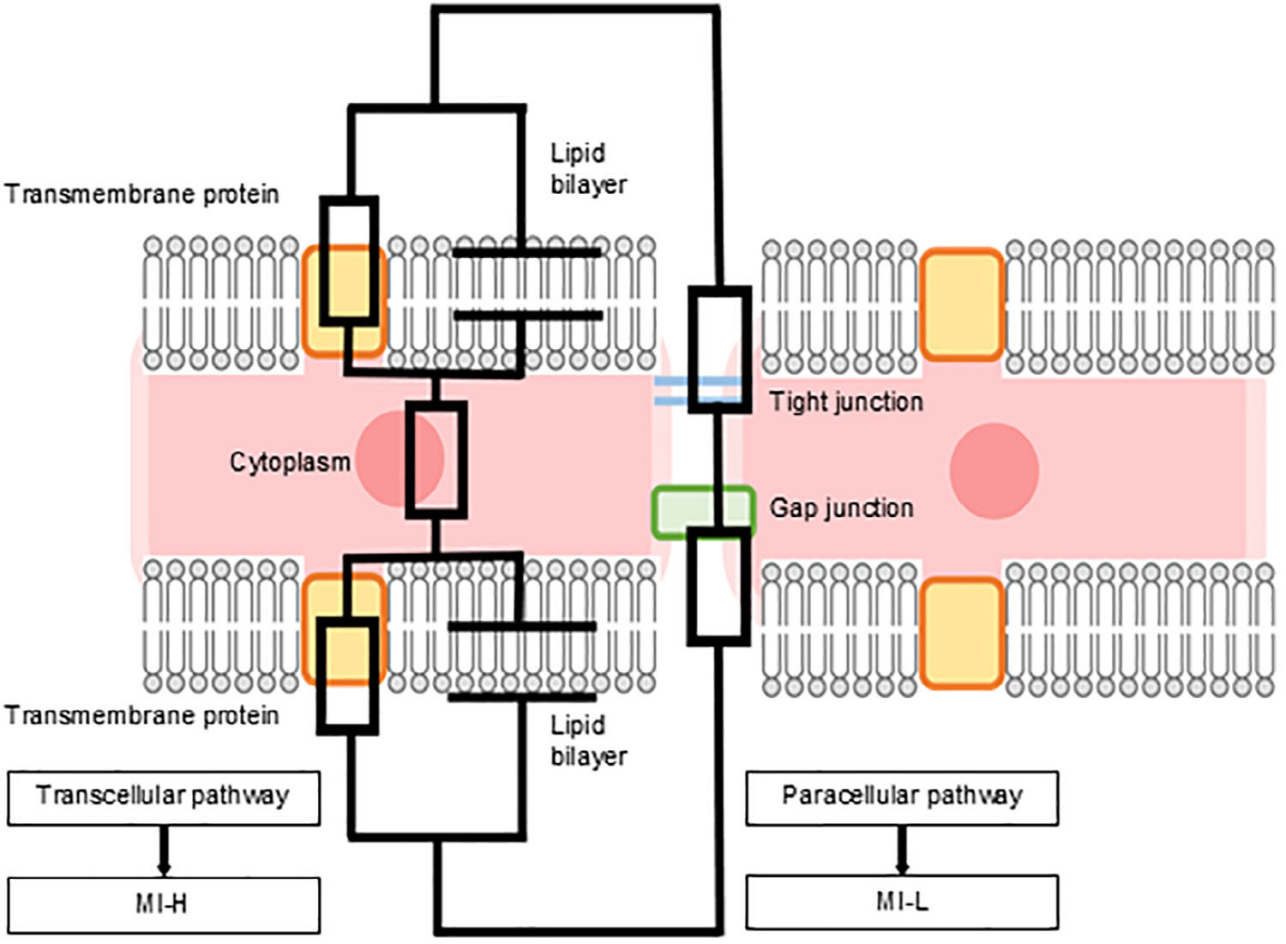
Schematic diagram of the cellular electrical circuit. This diagram displays electrical pathways within the epithelial layer. MI‐H reflects the transcellular pathway, whereas MI‐L reflects the paracellular pathway. MI‐H, mucosal impedance under high‐frequency currents; MI‐L, mucosal impedance under low‐frequency currents.

MI was measured using a TCM (AS‐TC100; Asch Japan Co., Ltd., Tokyo, Japan). Alternating currents at 320 Hz and 30.7 kHz were applied at a constant 12.5 mV voltage. High‐frequency currents mainly flow through the transcellular pathway, whereas low‐frequency currents primarily flow through the paracellular pathway. Accordingly, MI‐L reflects the paracellular pathway, and MI‐H reflects the transcellular pathway. Total transepithelial impedance can be represented by transcellular and paracellular impedances in parallel. Transcellular impedance consists of three impedance elements: resistance and capacitance parallel to the apical membrane, cytoplasmic resistance, and resistance and capacitance parallel to the basolateral membrane. Paracellular impedance can be described by two series components, namely junction resistance and intercellular gap resistance (Figure 1). The resistance of the intercellular gap is typically considerably lower than that of the junction [13].

### 2.3 Measurement of MI

ESD was conducted using upper gastrointestinal endoscopes (GIF‐H290T and GIF‐H260; Olympus, Tokyo, Japan). Submucosal injection was administered with a 23‐gauge injection needle using a solution of hyaluronic acid mixed with saline containing indigo carmine and epinephrine. A flush knife (Fujifilm, Tokyo, Japan) and hemostatic forceps (Radial Jaw 4 Hot Biopsy Forceps; Boston Scientific, Tokyo, Japan) were used. The high‐frequency unit (VIO3; Erbe, Tübingen, Germany) was set as follows: ENDO CUT I for mucosal incision, forced coagulation for submucosal dissection, and soft coagulation for hemostasis.

**FIGURE 2:**
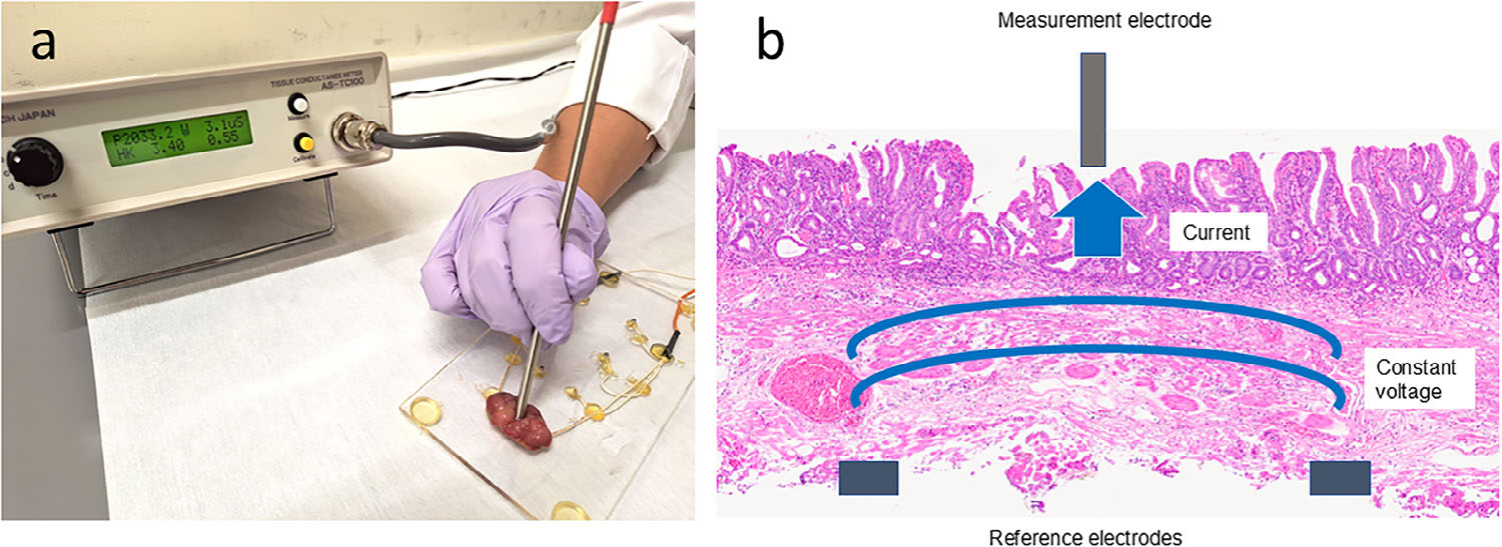
Schema illustration of MI measurement in ESD specimens. (a) The ESD‐resected specimen was placed on the reference electrodes with the resection surface downward. The mucosal surface of the ESD‐resected specimen was touched with the measurement electrode. (b) A constant voltage of 12.5 mV was applied to the submucosal layer, and alternating currents at 320 Hz and 30.7 kHz through the mucosa.

MI was measured in freshly resected ESD specimens. Immediately following resection, each specimen was placed on the reference electrode with the resection surface downward. The mucosal surface was gently contacted with the measurement electrode (a 2.5‐mm‐diameter catheter with a stick‐shaped electrode sensor at the tip) for 2–3 s. MI was recorded at both the main tumor lesion and the surrounding non‐neoplastic mucosa (Figure 2). Three consecutive measurements were obtained at each site, and the median value was used for analysis. Measurement sites were documented based on the circumferential markings. The specimen was then sectioned through the documented measurement sites so that the histological sections corresponded as closely as possible to the MI measurement sites.

### 2.4 Histopathological Evaluation and Immunohistochemical Staining

Histopathological evaluation, including tumor invasion and ulceration, was conducted by two experienced pathologists according to the Japanese Classification of Gastric Carcinoma. The evaluated items were scored as follows:

- Tumor invasion: pT1a for invasion confined to the mucosa and/or muscularis mucosa, and pT1b for invasion into the submucosa.
- Ulcer status (pUL): pUL0 when ulceration or ulcer scars were absent, and pUL1 when ulceration or ulcer scars were present.
- Macroscopic type (Type 0): Superficial flat tumors with or without minimal elevation or depression; Type 0‐I: protruded type; Type 0‐IIa: superficial elevated type; Type 0‐IIb: flat type; Type 0‐IIc: superficial depressed type; Type 0‐III: excavated type.

Atrophy and intestinal metaplasia were histologically evaluated using hematoxylin and eosin‐stained specimens.

**FIGURE 3:**
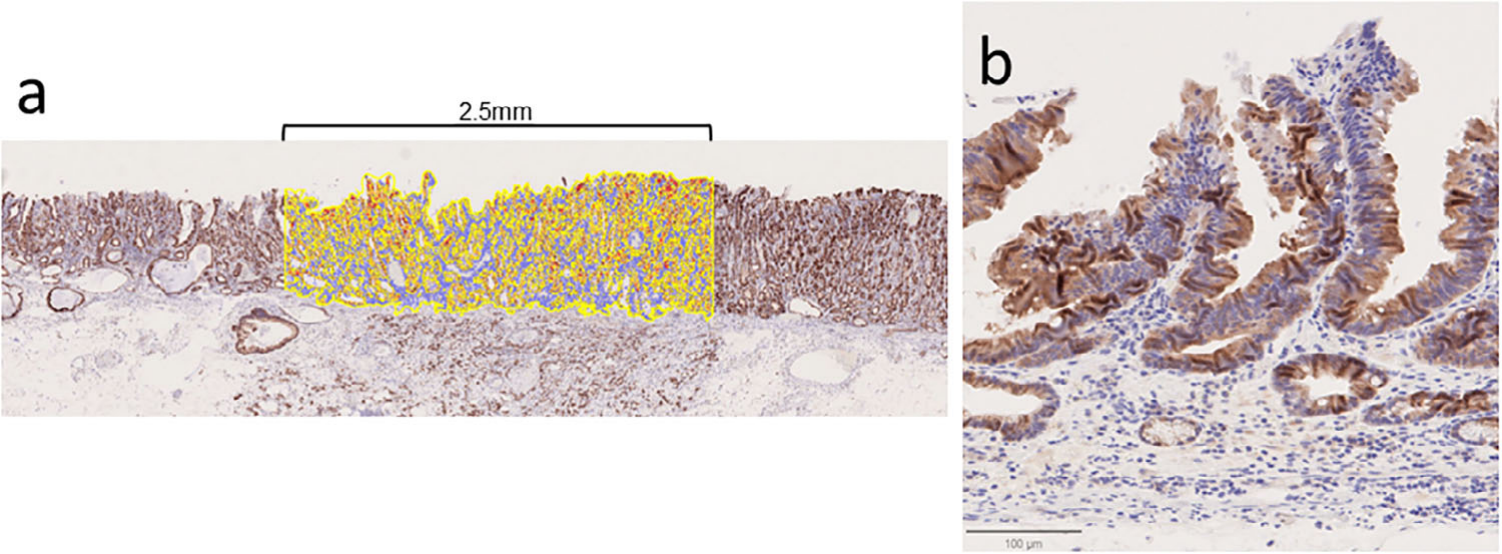
Representative images of H‐score analysis and immunohistochemical staining. (a) Representative image showing H‐score analysis using QuPath. A 2.5‐mm‐wide mucosal region corresponding to the MI measurement site was analyzed using the Positive Cell Detection algorithm. (b) Representative immunohistochemical staining of claudin‐18.

Immunohistochemical staining was performed using an automated system (BOND‐MAX; Leica Biosystems). Antigen retrieval was conducted via heat‐induced epitope retrieval using Tris‐EDTA buffer (pH 9.0). Rabbit monoclonal anti‐claudin‐3 antibody [EPR19971] (1:150 dilution; ab214487, Abcam, Cambridge, UK), anti‐claudin‐4 antibody [EPRR17575] (1:500 dilution; ab240384, Abcam), and anti‐claudin‐18 antibody [34H14L15] 1:500 dilution; ab203563, Abcam) were used as primary antibodies. Primary antibody binding was detected using a Bond Polymer Refine Detection kit (Leica Biosystems, Newcastle, UK) according to the manufacturer's protocol. The slides were scanned using a pathology slide scanner (NanoZoomer S210; Hamamatsu, Japan). The histological section containing the MI measurement site was selected for immunohistochemical analysis. All cells within a 2.5‐mm mucosal region corresponding to the MI measurement site were manually annotated and analyzed using the Positive Cell Detection algorithm in QuPath software (version 0.5.1). H‐scores were calculated by multiplying the percentage of cells stained at each intensity level (0, 1+, 2+, and 3+) by the corresponding intensity score and summing the values, yielding a score ranging from 0 to 300, which was used for analysis (Figure 3).

### 2.5 Statistical Analysis

Categorical variables are presented as frequencies, and continuous variables as medians and interquartile ranges (IQRs). The Mann–Whitney U test and one‐way analysis of variance were used for group comparisons. In the multivariable linear regression analysis, MI‐L was the dependent variable, while tumor invasion, ulcer status, macroscopic type, and the H‐scores for each claudin were included as independent variables. All statistical analyses were performed using EZR (Saitama Medical Center, Jichi Medical University, Saitama, Japan), a graphical user interface for R (R Foundation for Statistical Computing, Vienna, Austria). EZR is a modified version of R Commander that incorporates statistical functions frequently used in biostatistics [14]. A *p*‐value of < 0.05 was considered statistically significant.

## 3 Results

### 3.1 Patient Characteristics

**FIGURE 4:**
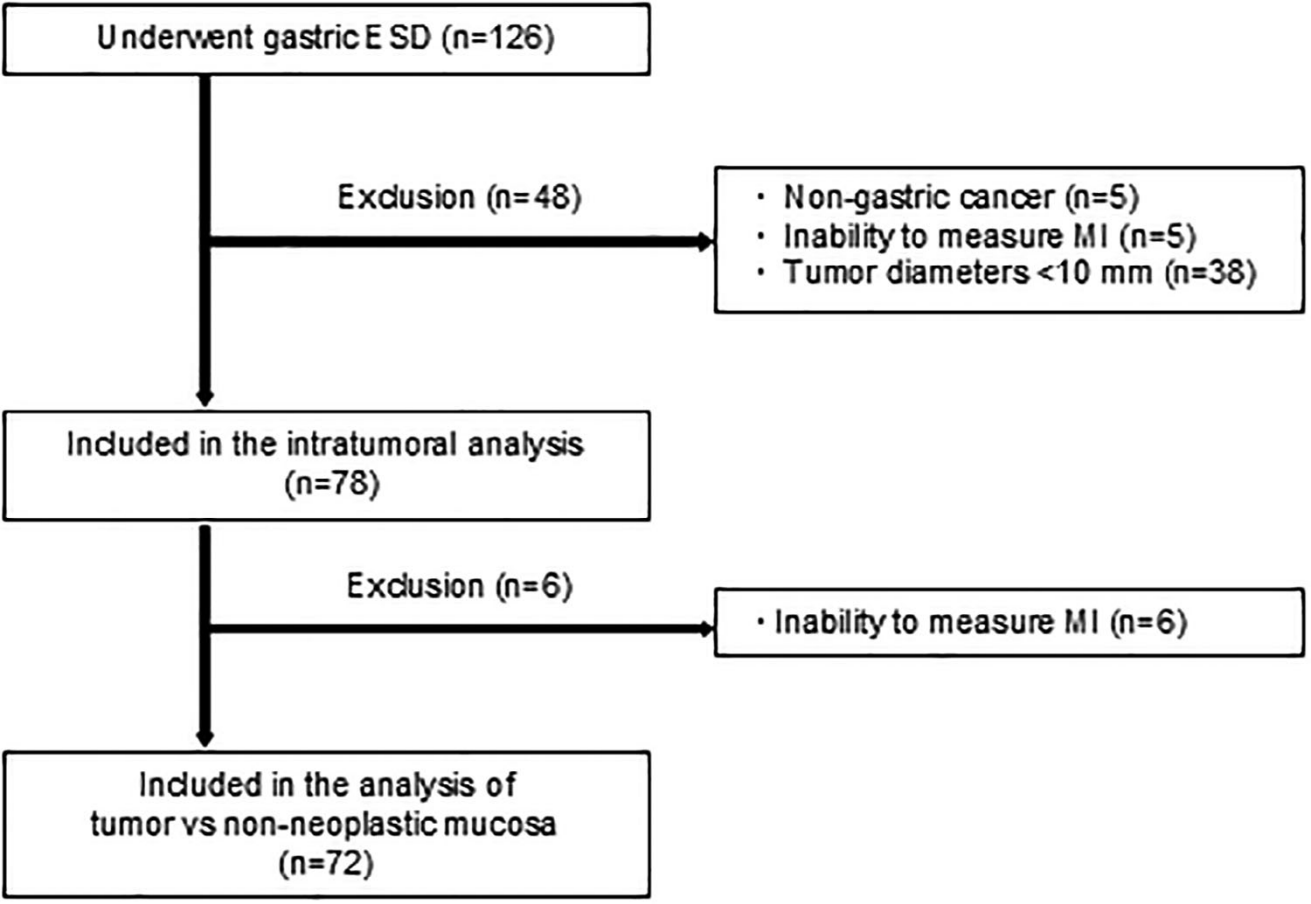
Flow diagram of study enrollment.

A total of 126 ESD procedures were performed for gastric neoplasms during the study period. Five cases diagnosed with non‐gastric cancer, five cases in which accurate measurements could not be obtained, and 38 cases with tumor diameters < 10 mm were excluded. Overall, 78 lesions from 73 patients were analyzed (Figure 4). Baseline characteristics are shown in Table 1. The median age was 76 years (IQR 72–81), and 54 patients (74.0%) were men. Thirty‐six patients (49.3%) were receiving PPI or PCAB therapy. Seven patients (9.6%) had a smoking history, and 25 (34.3%) had a drinking history. Regarding *H. pylori* status, 8 patients (11.0%) had current infection, 64 (87.7%) had past infection, and 1 (1.4%) was uninfected.

**TABLE 1:** Baseline characteristics.

Parameter	*n*	
Total patients	73	
Age, years (range)	76	(72–81)
Sex		
Male, *n* (%)	54	(74.0)
Female, *n* (%)	19	(26.0)
PPI/PCAB		
Taking, *n* (%)	36	(49.3)
Not taking, *n* (%)	37	(50.7)
Smoking habit, *n* (%)	7	(9.6)
Drinking habit, *n* (%)	25	(34.3)
Helicobacter pylori infection status		
Current infection	8	(11.0)
Past infection	64	(87.7)
Uninfected	1	(1.4)
Patients with two lesions	5	(6.8)
Total lesions	78	
Procedure times (min), median (range)	104	(62.75–145.25)
En bloc resection, *n* (%)	77	(98.7)
Tumor depth		
pT1a, *n* (%)	66	(84.6)
pT1b, *n* (%)	12	(15.4)
Ulceration		
pUL0, *n* (%)	67	(85.9)
pUL1, *n* (%)	11	(14.1)
Location		
U, *n* (%)	24	(30.8)
M, *n* (%)	42	(53.9)
L, *n* (%)	12	(15.4)
Gross types		
0‐I, *n* (%)	1	(1.28)
0‐IIa, *n* (%)	37	(47.4)
0‐IIb, *n* (%)	6	(7.7)
0‐IIc, *n* (%)	30	(38.5)
Others, *n* (%)	4	(5.1)
Tumor diameters (mm), median (range)	18	(10–25)
Histology		
Differentiated adenocarcinoma, *n* (%)	76	(97.4)
Undifferentiated adenocarcinoma, *n* (%)	2	(2.6)
Perilesional atrophy, *n* (%)	76	(97.4)
Perilesional intestinal metaplasia, *n* (%)	76	(97.4)

Abbreviations: P‐CAB, potassium‐competitive acid blocker; PPI, proton pump inhibitor.

Based on tumor depth, 66 (85.9%) and 12 (15.4%) lesions were classified as pT1a and pT1b, respectively. Regarding ulcer status, 67 (85.9%) and 11 (14.1%) lesions were classified as pUL0 and pUL1, respectively. Macroscopic types were as follows: 1 (1.3 %), 37 (47.4 %), 6 (7.7 %), and 30 (38.5 %) lesions were Types 0‐I, 0‐IIa, 0‐IIb, and 0‐IIc, respectively. Regarding tumor location, 12 (15.4%), 42 (53.9%), and 24 (30.8%) lesions were in the upper, middle, and lower thirds of the stomach, respectively. Perilesional atrophy and intestinal metaplasia were both observed in 76 lesions (97.4%).

### 3.2 Tumor Versus Non‐neoplastic MI

Among the 78 lesions evaluated, six were excluded because the non‐neoplastic mucosa could not be measured. When comparing MI between tumors and surrounding non‐neoplastic mucosa, MI‐H in tumors was significantly lower than that in the non‐neoplastic mucosa. No significant difference was observed between the MI‐L of the tumor and non‐neoplastic mucosa (Table 2).

**TABLE 2:** Mucosal impedance of tumors and non‐neoplastic mucosa.

		MI‐H			MI‐L		
	*n*	median (IQR)		*p*‐value	median (IQR)		*p*‐value
Tumor	72	94.2	(86.7–105.0)	0.002	8.8	(7.0–11.4)	0.305
Non‐neoplastic mucosa	72	101.0	(94.2–105.0)		9.3	(7.6–11.5)	

Abbreviations: IQR, interquartile range; MI‐H, mucosal impedance under high‐frequency currents; MI‐L, mucosal impedance under low‐frequency currents.

To further evaluate the impedance differences between tumors and surrounding mucosa, the ratios of MI‐H and MI‐L were defined as rMI‐H and rMI‐L, respectively. No significant differences in impedance ratio were observed between pT1a and pT1b, pUL0 and pUL1, or Type 0‐IIa and 0‐IIc lesions (Table 3).

**TABLE 3:** Ratio of mucosal impedance between tumor and non‐neoplastic mucosa.

		rMI‐H			rMI‐L		
	*n*	median (IQR)		*p*‐value	median (IQR)		*p*‐value
Tumor depth							
pT1a	62	0.95	(0.84–1.02)	0.328	1.02	(0.78–1.29)	0.076
pT1b	10	0.80	(0.68–1.06)		0.62	(0.54–1.07)	
Ulceration							
pUL0	65	0.93	(0.83–1.09)	0.150	0.95	(0.76–1.32)	0.125
pUL1	7	0.86	(0.71–0.91)		0.76	(0.56–1.00)	
Gross types							
0‐IIa	34	1.02	(0.81–1.17)	0.126	1.07	(0.74–1.47)	0.249
0‐IIc	29	0.90	(0.80–1.00)		0.97	(0.72–1.18)	

Abbreviations: IQR, interquartile range; rMI‐H, mucosal impedance ratio between tumor and non‐neoplastic mucosa under high‐frequency currents; rMI‐L, mucosal impedance ratio between tumor and non‐neoplastic mucosa under low‐frequency currents.

### 3.3 MI Within the Tumor

MI‐L in patients with pT1b was significantly lower than in patients with pT1a; however, no difference was observed in the MI‐H (Table 4). Similarly, MI‐L in patients with pUL1 was significantly lower than in patients with pUL0, with no difference in the MI‐H. No significant differences in MI were observed according to PPI use, tumor location, *H. pylori* status (current vs. past infection), smoking habit, or drinking habit. However, because the background mucosa of almost all lesions showed atrophy and intestinal metaplasia, and only one lesion occurred in an *H. pylori*‐uninfected stomach, the influence of these background mucosal factors could not be adequately evaluated. Moreover, no correlation was observed between MI and procedure time (MI‐H vs. resection time: *r* = 0.122, *p* = 0.289; MI‐L vs. resection time: *r* = 0.173, *p* = 0.129).

**TABLE 4:** Mucosal impedance within tumor.

		MI‐H		MI‐L	
	*n*	median (IQR)	*p*‐value	median (IQR)	*p*‐value
Tumor depth					
pT1a	58	95.6 (89.3–108.6)	0.243	9.7(8.0–11.8)	0.021
pT1b	9	85.7 (83.2–102.2)		6.9 (6.5–7.7)	
Ulceration					
pUL0	58	95.6 (89.3–108.6)	0.097	9.7 (8.0–11.8)	0.030
pUL1	8	89.4 (84.0–96.6)		7.5 (6.9–7.9)	
Gross types					
0‐IIa	37	96.1 (92.8–112.7)	0.053	9.8 (8.4–12.3)	0.031
0‐IIc	30	92.6 (86.7–99.7)		8.5 (6.7–9.8)	
Location					
U	12	97.4 (88.7–107.3)	0.757	8.7 (6.9–10.6)	0.865
M	42	94.8 (88.6–101.8)		8.8 (7.5–11.8)	
L	24	93.7 (83.1–106.8)		9.0 (7.0–11.4)	
*Helicobacter pylori* infection status					
Current infection	9	94.8 (87.5–103.8)	0.918	8.9 (7.1–11.0)	0.849
Past infection	68	93.9 (88.4–106.4)		8.6 (6.8–12.0)	
PPI/PCAB					
Taking	41	97.1 (87.7–106.4)	0.524	9.7 (7.1–11.7)	0.352
Not taking	37	93.6 (88.4–99.1)		8.5 (7.1–11.0)	
Smoking habit					
Yes	8	94.9 (92.3–99.8)	0.954	8.9 (7.1–11.2)	0.889
No	70	94.8 (87.1–105.3)		9.3 (7.1–11.9)	
Drinking habit					
Yes	28	94.8 (85.4–105.7)	0.872	8.6 (7.1–11.8)	0.700
No	50	95.1 (88.5–103.2)		9.3 (7.1–11.0)	

Abbreviations: IQR, interquartile range; MI‐H, mucosal impedance under high‐frequency currents; MI‐L, mucosal impedance under low‐frequency currents.

### 3.4 Immunohistochemical Staining for Claudin‐3, ‐4, and ‐18

In the tumor depth analysis, the H‐score of claudin‐4 was significantly lower in pT1b than in pT1a. No significant differences were observed in the H‐score of claudin‐3 or ‐18 between pT1a and pT1b. In the pUL analysis, no significant differences were observed in H‐scores of claudin‐3, ‐4, and ‐18 between pUL0 and pUL1 cases (Table 5).

**TABLE 5:** H‐score of claudin‐3, ‐4, and ‐18 according to tumor depth and ulcerative findings.

		Claudin‐3		Claudin‐4		Claudin‐18	
	*n*	median (IQR)	*p*‐value	median (IQR)	*p*‐value	median (IQR)	*p*‐value
Tumor depth							
pT1a	58	1.3(0.2–6.3)	0.157	111.9(56.9–142.0)	0.016	99.4(60.2–126.9)	0.100
pT1b	9	0.3(0.1–0.8)		53.6(20.9–91.5)		71.0(37.6–90.6)	
Ulceration							
pUL0	58	1.2(0.2–6.3)	0.331	111.9(56.9–142.0)	0.449	99.4(60.2–126.9)	0.658
pUL1	8	4.8(0.4–21.3)		103.5(44.5–128.2)		92.1(73.5–115.7)	

Abbreviation: IQR, interquartile range.

**FIGURE 5:**
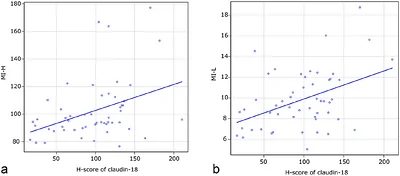
Correlation between H‐score of Claudin‐18 and MI: (a) H‐score of claudin‐18 versus MI‐H. (b) H‐score of claudin‐18 versus MI‐L.

No significant correlations were observed between H‐scores of claudin‐3 or claudin‐4 and MI (claudin‐3 vs. MI‐H: *r* = −0.013, *p* = 0.93; claudin‐3 vs. MI‐L: *r* = −0.208, *p* = 0.139; claudin‐4 vs. MI‐H: *r* = −0.243, *p* = 0.083; claudin‐4 vs. MI‐L: *r* = −0.155, *p* = 0.271). In contrast, claudin‐18 showed significant positive correlations with MI (claudin‐18 vs. MI‐H: *r* = 0.382, *p* = 0.005; claudin‐18 vs. MI‐L: *r* = 0.423, *p* = 0.002) (Figure 5). Furthermore, multivariable linear regression analysis demonstrated that, among the explanatory variables—including tumor invasion, ulcer status, macroscopic type, and the H‐scores for each claudin‐ only the H‐score of claudin‐18 was identified as an independent associated factor (*β* = 0.023, 95% CI 0.006–0.040, *p* = 0.007) (Table 6).

**TABLE 6:** Multivariable linear regression analysis of factors associated with MI‐L.

Variables	*β*	95%CI	*p*‐value
Tumor depth	−1.787	−4.115–0.541	0.130
pUL	−2.264	−4.742–0.213	0.073
Macroscopic type	−0.5119	−1.613–0.590	0.357
Caludin‐3	0.049	−0.065–0.163	0.391
Claudin‐4	−0.008	−0.024–0.007	0.258
Claudin‐18	0.023	0.006–0.040	0.007

Abbreviations: β, unstandardized regression coefficient; CI, confidence interval.

## 4 Discussion

This study showed that the MI‐H in the tumor was significantly lower than that in the non‐neoplastic mucosa. More importantly, the histological characteristics also influenced MI‐L. The MI‐L of EGC lesions was lower in pT1b or pUL1 lesions, suggesting early abnormalities in the paracellular pathway. Endoscopic imaging can distinguish tumor from non‐tumor mucosa; in contrast, MI may reflect histological tumor characteristics.

MI in the paracellular pathway is predominantly influenced by tight junctions [12]. Therefore, the low MI‐L in tumors reflects disruption of mucosal barrier function. Although claudin‐3, ‐4, and ‐18 are all tight junction proteins, they have distinct biological functions in the gastric epithelium [10]. From our multivariable analysis, the H‐score of claudin‐18 remained independently associated with MI‐L after adjustment for submucosal invasion, pUL, macroscopic type, and the H‐score of claudin‐3 and ‐4. This finding suggests that decreased claudin‐18 expression contributes to the decreased MI‐L, independent of submucosal invasion and ulceration. Unlike claudin‐3 and ‐4 “intestinal‐type claudins”, claudin‐18 “stomach‐type claudin” is the predominant claudin expressed and regulates the stomach‐specific properties of the paracellular barrier. A previous study using claudin‐18 knockout mice showed that loss of claudin‐18 leads to paracellular H^+^ leakage, mucosal inflammation, and atrophic gastritis [15]. Therefore, reduced claudin‐18 expression may contribute to the decrease in MI‐L.

Clinically, accurate assessment of the depth of invasion and UL in EGC is essential for determining appropriate treatment; however, these assessments may vary among endoscopists. Tumor size >30 mm, lymphatic invasion, venous invasion, positive vertical margin, and submucosal invasion ≥ 500 µm have been associated with lymph node metastasis [16]. Therefore, assessment of these factors is clinically important. Moreover, evaluation of tumor depth and UL is important for determining the technical feasibility of ESD. The depth of invasion and UL in gastric cancers are generally evaluated using white‐light imaging, and endoscopic ultrasonography can be useful for assessing the depth of invasion [17]. However, accurate evaluation of invasion depth and UL remains challenging in EGC. Studies have reported that the positive predictive value of tumor depth assessment using white‐light imaging ranges from 69.3% to 82.0% and from 60.2% to 71.9% for pT1a and pT1b cases, respectively [18, 19]. Moreover, the positive predictive value of the preoperative evaluation of ulceration in EGC is relatively low (61.3%) [20]. Furthermore, accurate evaluation is difficult in cases of pT1b or pUL1. In our study using resected specimens, MI values were associated with histological features such as submucosal invasion and ulceration.

Contrary to our hypothesis, the MI‐H did not exhibit significant differences between submucosal invasion and UL. Because EGC demonstrates various histological types and contains a substantial proportion of stromal cells, tumor cell proliferation does not necessarily correlate with cellular density, which may explain the absence of significant differences in MI‐H. Regarding macroscopic type, both MI‐H and MI‐L tended to be higher in Type 0‐IIa lesions than in Type 0‐IIc lesions, suggesting that a greater abundance of structural components in Type 0‐IIa lesions may contribute to the higher MI values. No significant associations were identified between MI and PPI/P‐CAB use, tumor location, *H. pylori* status (current vs. past infection), smoking habit, drinking habit, or procedure time. However, because multiple factors influence gastric mucosa, these relationships may not have been detected in univariate analysis. Although the amount of submucosal tissue was not calculated, the measurement principle suggests that submucosal thickness is unlikely to substantially influence the obtained MI values because the small measurement electrode allows the measurement to mainly reflect tissue close to the electrode surface, with limited contribution from deeper tissue layers [14].

This study has some limitations. First, as measurements were performed on resected specimens, MI may have been influenced by procedural factors such as submucosal injection and thermal injury, as well as by loss of cell viability and absence of blood flow. Although multiple factors, such as *H. pylori* infection status, PPI/P‐CAB use, and gastritis, can influence the background gastric mucosa, multivariable analysis was performed only for selected clinicopathological factors and claudin H‐scores. In addition, because the study population was highly homogeneous with respect to background mucosal status, the effects of *H. pylori* infection, atrophy, and intestinal metaplasia on MI could not be assessed. Finally, although the specimen was sectioned through the documented measurement site, the histological section may not have completely matched the MI measurement site.

To overcome these limitations, further studies involving larger samples and multivariate analyses are required. Additionally, measuring non‐neoplastic mucosa before treatment will help clarify the influence of background gastric mucosa on MI. Future in vivo endoscopic studies are also warranted to evaluate MI directly.

In conclusion, MI‐L in resected specimens was independently associated with claudin‐18 expression and correlated with submucosal invasion, ulcer status, and macroscopic type. MI has the potential to measure non‐surface changes in EGC that cannot be observed via endoscopy.

## Authors Contributions

**Study conception and design**: Kaede Miyashiro and Yohei Sawada. **Manuscript preparation**: Kaede Miyashiro, Yohei Sawada, and Hidezumi Kikuchi. **Endoscopic treatment**: Kaede Miyashiro, Yohei Sawada, Hidezumi Kikuchi, Taka Asari, Saki Nakano, Tatsuta Tetsuya, and Daisuke Chinda. **Measurement of mucosal impedance**: Kaede Miyashiro, Yohei Sawada, and Hidezumi Kikuchi. **Histopathological evaluation**: Shintaro Goto and Tadashi Yoshizawa. **Critical revision of the article for important intellectual content**: Hirotake Sakuraba. **Final approval of the article**: all authors

## Conflicts of Interest

The authors declare no conflicts of interest.

## Funding

The authors have nothing to report.

## Ethics Statement

This study was approved by the ethics committee of Hirosaki University Graduate School of Medicine (No. 2023–024). Written informed consent was obtained from all patients.
